# Medial single-window approach to the elbow: a triceps-on technique that does not violate the olecranon bursa

**DOI:** 10.1016/j.xrrt.2025.08.016

**Published:** 2025-09-09

**Authors:** Gregory Bain, Simon Bellringer, John White, Jonathan P. Evans

**Affiliations:** a Department of Orthopaedic Surgery, Flinders Medical Centre, Adelaide, Australia; b Flinders University, Adelaide, Australia; c University Hospitals Sussex NHS Foundation Trust, Sussex, UK; d University of Exeter Medical School, Royal Devon University Healthcare NHS Foundation Trust, Exeter, UK

**Keywords:** Elbow, Surgical approach, Surgical technique, Elbow arthroplasty, Elbow replacement, Medial shotgun, Hemiarthroplasty

The surgical approach to the elbow must balance adequate exposure with judicious preservation of anatomical structures. This is particularly critical when undertaking elbow arthroplasty, a rewarding intervention in a carefully selected cohort of patients, with indications including painful arthritis, post-trauma sequalae, and increasingly in the treatment of unreconstructible distal humeral fractures in the elderly.^5^ Various surgical approaches have been described in the literature, and techniques continue to evolve to optimize functional outcomes and implant longevity while minimizing complications such as infection, triceps deficiency, instability, and wound healing issues. In particular, concerns persist regarding healing of the wound under tension on the extensor surface, potential compromise of the olecranon bursa and the devastating outcome of infection.

Most surgical approaches to the elbow utilize a midline posterior incision with full-thickness skin flaps and early identification of the ulnar nerve.^5^ Traditional techniques relied on a ‘triceps-off’ approach, either through a triceps turndown or subperiosteal dissection from the triceps insertion. However, these approaches are associated with postoperative triceps failure and reduced strength.^12^ There has, therefore, been a shift toward ‘triceps-on’ approaches, where the insertion of the triceps is preserved and either a single or dual working windows are created around the muscle.^2^^,^^3^

The dual window approaches, such as the Global approach^2^^,^^14^^,^^18^ and Alonso Llames approach,^1^ involve dissection of the lateral and medial humeral supracondylar ridges. Distally, the Global approach utilizes the Kocher interval on the lateral side,^24^ and on the medial side elevation of the flexor carpi ulnaris (FCU) from the proximal ulna, to expose the medial ligament and joint.^18^ The lateral paraolecranon approach^26^ also employs a midline posterior approach and medial window but differs laterally where the triceps is split in line with the lateral edge of the ulna, and exposure of the distal humerus is gained through this interval.

Single lateral window approaches, an evolution of the Kocher approach that utilize the interval between anconeus and extensor carpi ulnaris, have also been described for elbow arthroplasty.^24^ In our cadaveric trials of the single lateral window approach, we found that releasing the common extensor origin, performing a lateral ligamentous release, and hinging the joint open on the medial collateral ligament (MCL) had a significant limitation: it did not allow for safe exposure and protection of the ulnar nerve without a secondary medial approach. This limitation negates many of the potential advantages.

This study investigates the hypothesis that a medial single-window approach, utilizing a triceps-on technique, may provide a safe and adequate exposure of the elbow joint while reducing the potential complications associated with dissection through the olecranon bursa. In principle, a single-window medial approach enables early identification of the ulnar nerve and allows for clear visualization of the humeral and ulnar articular surfaces. This is achieved by releasing the MCL and the anterior and posterior capsular structures, followed by a surgical dislocation hinging on the intact lateral ligament complex. By avoiding violation of the olecranon bursa and preserving lateral structures, this approach offers a viable alternative that ensures sufficient visualization for total elbow arthroplasty (TEA) while minimizing postoperative complications and allowing early safe mobilization.

## Methods

The development of the surgical technique was undertaken through cadaveric assessment. Twenty fresh frozen cadavers were utilized, and the feasibility of the surgical approach, safety, exposure, and implantation of prosthesis was critically appraised by 4 fellowship-trained upper limb orthopedic surgeons. All cadaveric assessment was undertaken in accordance with the South Australia Transplantation and Anatomy Act 1983 and performed in a registered facility.

Subsequent to the technique development, the approach has been utilized in 10 clinical cases of TEA. In all cases, the Stryker Latitude linked total elbow replacement was used by the senior authors.

### Technique

We describe the surgical technique that we recommend, based on the lessons learnt from the cadaveric and clinical cases.

#### Set up and superficial dissection

**Figure 1:**
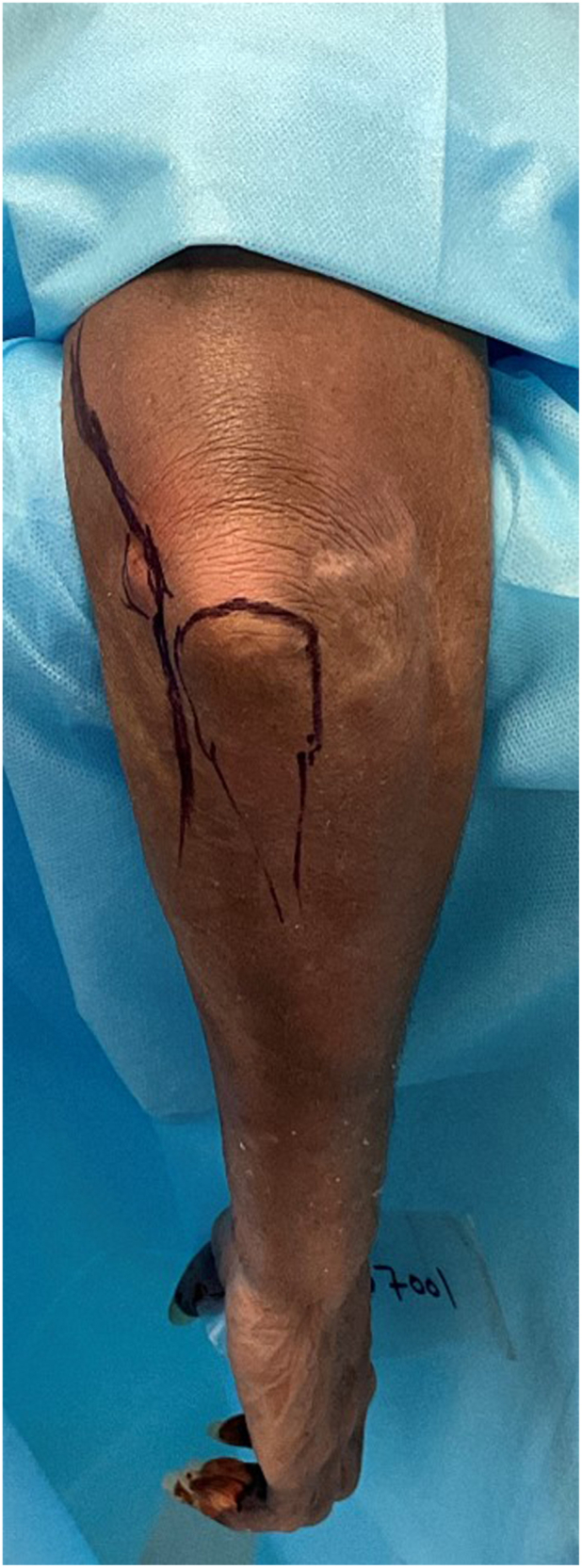
Cadaveric specimen of a right elbow positioned over an elbow gutter. Skin marking to show the olecranon process and medial epicondyle. The incision is marked, 10 cm along the medial border of the triceps, and 5 cm along the medial subcutaneous border of the ulna. Copyright Dr. Gregory Bain 2025.

**Figure 2:**
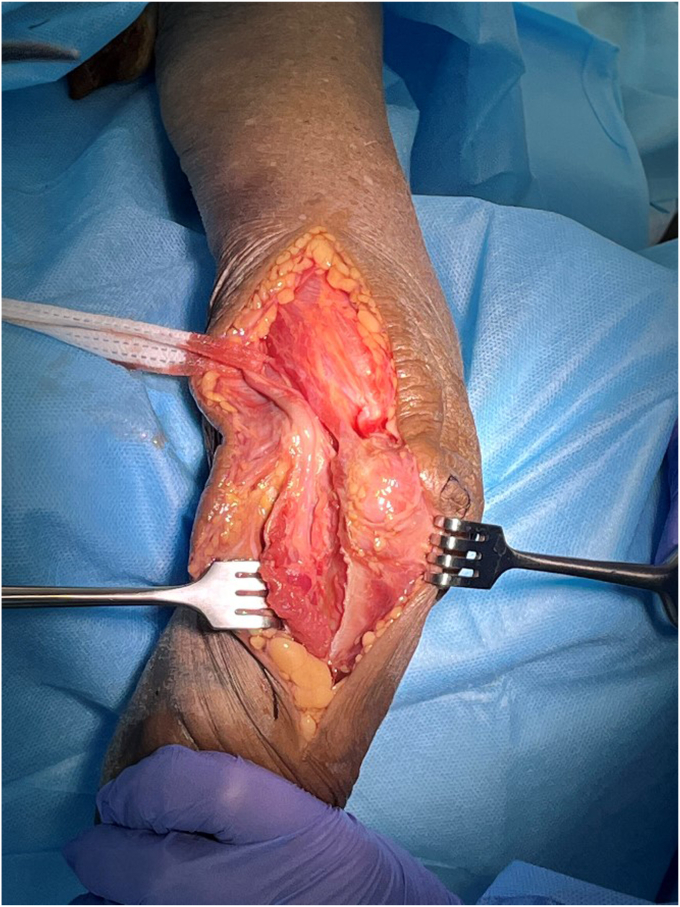
Superficial dissection: The deep fascia is incised on the medial border of the triceps, and the subcutaneous border of the ulna. The large muscular anterior flap includes the ulnar nerve with its soft tissue envelope—brachialis, biceps, and FCU. The olecranon and its bursa are not exposed. In this cadaveric photo, we have exposed the medial aspect of the triceps for clarity, but in clinical practice only the medial edge of triceps is seen, to reduce the dead space in the wound. Copyright Dr. Gregory Bain 2025. *FCU*, flexor carpi ulnaris.

The patient is positioned in the supine position, with the arm on an arm board (Figure 1, Figure 2 and Figure 1, Figure 2). An alternative is the lateral decubitus position, with the arm supported on an elbow gutter, positioned so that the elbow hangs at 90° (Fig. 1). A sterile tourniquet is placed on the upper arm away from the surgical site. Alternatively, the procedure can be performed without a tourniquet, using 1 g intravenous tranexamic acid administered at anesthetic induction and adrenaline injection 1 ml of 1/1000, diluted in 50 ml of normal saline, infiltrated into the medial soft tissues. A 15 cm incision is made over the posteromedial aspect of the elbow. Proximally, this is along the medial border of the triceps for 10 cm, extending to the medial aspect of the olecranon, and then extending 5 cm along the medial border of the subcutaneous ulna. The subcutaneous fat and deep fascia are incised directly over the medial border of the triceps muscle. The cubital retinaculum and the FCU muscle are released from the subcutaneous border of the ulna. We leave a 5 mm cuff of the FCU fascia on the proximal ulna, for later surgical repair (Fig. 2). We perform most of the dissection with sharp dissection or fine-point cautery. At no time during the procedure, is the olecranon bursa, or its overlying skin violated.

#### Medial exposure and ulnar nerve

At the proximal extent of the wound, the soft tissue is dissected from the medial border of the triceps, and the triceps is retracted posteriorly to expose the humerus (Fig. 2). The superficial and deep dissection on the medial border of the triceps is extended to the distal humerus and ulna. The cubital retinaculum and FCU muscle are released from the medial border of the proximal ulna, until the coronoid process is exposed.

By reflecting the soft tissues, on the medial border of the triceps, the ulnar nerve and it's perineural soft tissue envelope is maintained. Proximally the ulnar nerve remains with the brachialis and medial intramuscular septum. Distally, it lies on the deep surface of the FCU and the large anterior muscle flap. Any fibrous bands that cross the ulnar nerve are released. The vascularity of the ulnar nerve is maintained, as it is not dissected from its anterior soft tissues (Fig. 2).

#### Posterior exposure

**Figure 3:**
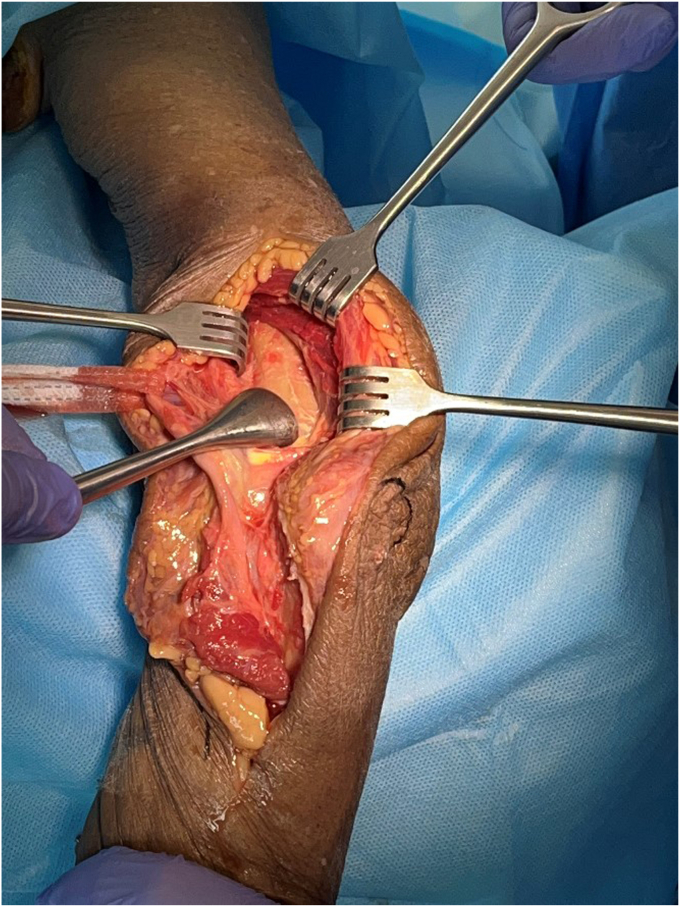
Deep posterior dissection: Subperiosteal release of the triceps from the posterior humerus. Copyright Dr. Gregory Bain 2025.

The triceps is released subperiosteally from the humerus across the full width of the shaft and distal humerus (Fig. 3). The posterior capsule and fat pad are excised.

**Figure 4:**
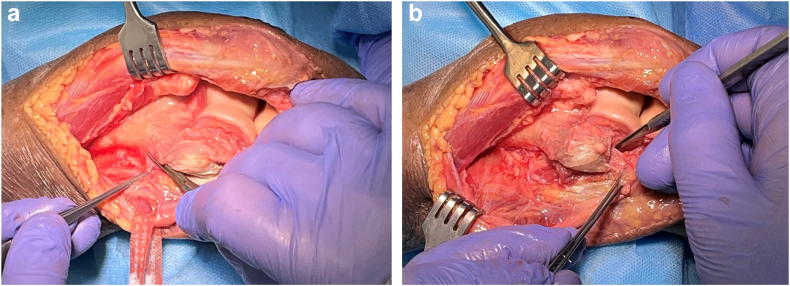
Medial release: (**a**) Release of the medial intermuscular septum from the humerus to allow access into the anterior compartment. Tape on the ulnar nerve. (**b**) The MCL is released from its humeral attachment. Copyright Dr. Gregory Bain 2025. *MCL*, medial collateral ligament.

The medial intermuscular septum is released from the medial humeral ridge, and the adjacent vessels cauterized (Fig. 4, *A*). This allows exposure of the anterior compartment.

**Figure 5:**
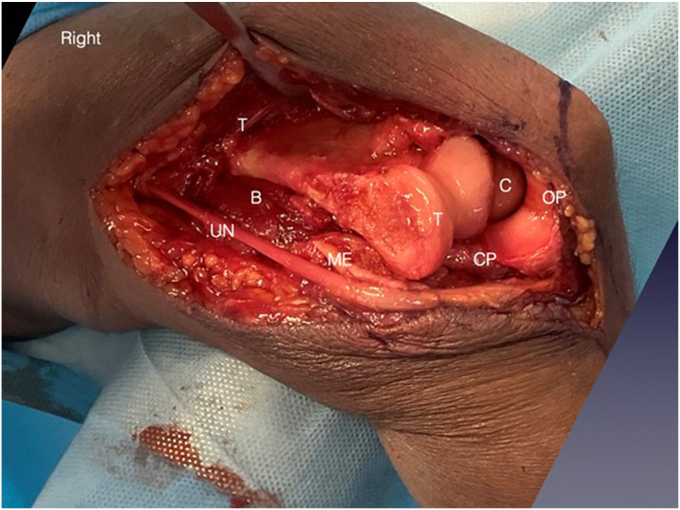
ME osteotomy. Note that the medial muscular flap includes the brachialis (B), medial intermuscular septum, ME, medial collateral ligament, ulnar nerve and the FCU muscle. This muscular flap is retracted together, to allow the humerus to dislocate medially. With the joint dislocated, the CP and olecranon process can be seen (OP). The osteotomy can be stabilized with sutures, screw or small plate. Copyright Dr. Gregory Bain 2025. *ME*, medial epicondyle; *CP*, coronoid process; *B*, brachialis; *OP*, olecranon process; *UN*, ulnar nerve; *T*, trochlear; *C*, capitellum; *FCU*, flexor carpi ulnaris.

The anterior and posterior bundles of the MCL are released from their humeral attachment. They can be tagged for subsequent repair (Fig. 4, *B*). An alternative is to perform a basal medial epicondyle osteotomy with an oscillating saw. This avoids the need to incise the MCL or release the common flexor origin from the medial epicondyle (Fig. 5).

**Figure 6:**
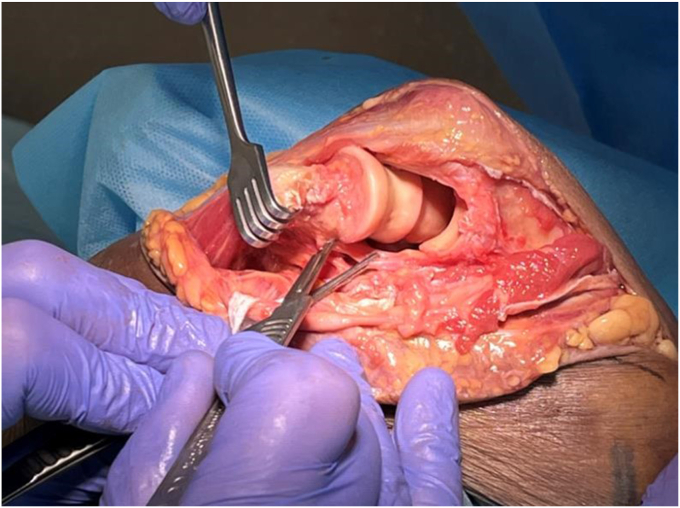
Anterior release: the forceps are holding the anterior capsule, which is being released from the distal humerus with the scalpel. Once the anterior and posterior capsule have been released, the distal humerus will prolapse through the medial single window. Copyright Dr. Gregory Bain 2025.

The brachialis is released across the full width of the shaft and distal humerus in the subperiosteal plane. The anterior capsule is excised (Fig. 6).

#### Dislocation

**Figure 7:**
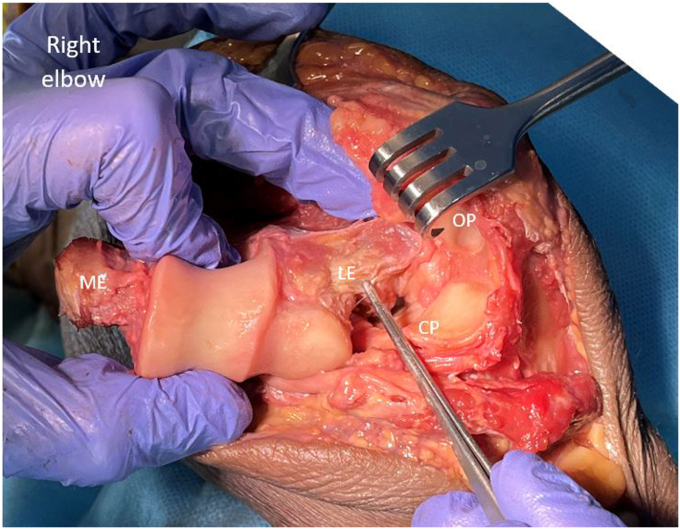
Lateral exposure: the capsular attachments to the posterior, medial and anterior distal humerus have been released, and the elbow is dislocated. The joint is now hinging on the lateral ligament complex (forceps), and the muscles of the lateral supracondylar ridge. We typically leave these intact, to optimize stability. The lateral ligament complex and supracondylar muscles can be release from the lateral humerus if extra exposure is required. Copyright Dr. Gregory Bain 2025. *OP*, Olecranon process; *CP*, Coracoid process; *ME*, Medial epicondyle; *LE*, Lateral epicondyle.

With careful retraction of the anterior flap–brachialis, FCU and ulnar nerve, a valgus force is placed on the elbow, to prolapse the distal humerus through the medial window (Supplementary Video S1 and 2, Fig. 7). To disengage the olecranon, the forearm is pronated, thereby subluxating and then dislocating the joint. A bone holding forceps, (e.g. Verbrugge), can be used to facilitate the manipulation and dislocation of the humerus. The elbow is now hinging on the lateral ligament complex (Fig. 7).

The opening of the joint from the medial side, for visualization of the distal humerus and proximal ulna, can be termed a ‘shotgun break-action’ maneuver, where the distal humerus is dislocated through this medial window. Posterior to the humerus are the triceps and ulna and anteriorly the brachialis, biceps, ulnar nerve, and common flexor muscles.

#### Lateral exposure

**Figure 8:**
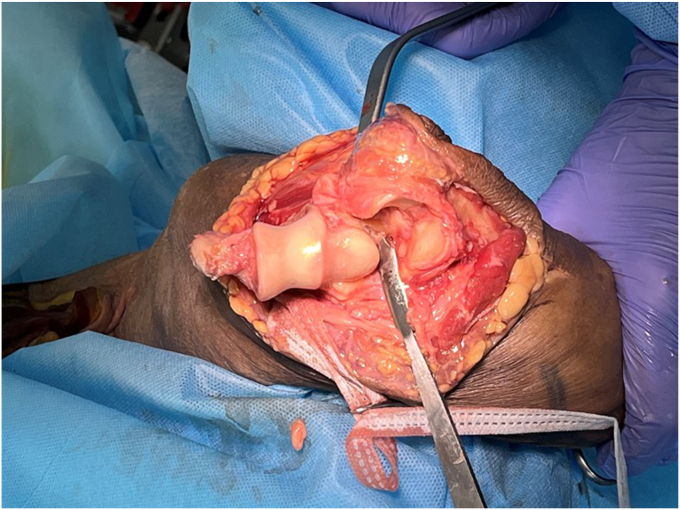
Joint dislocated. Note the position of the Hohmann retractor over the lateral ligament to maintain exposure of the entire articular surfaces of the distal humerus and proximal ulna. Behind the Hohmann retractor is the proximal surface of the radial head. To expose the radial neck and head, release the annular ligament from the sigmoid notch of the proximal ulna. Copyright Dr. Gregory Bain 2025.

A lateral joint synovectomy can be performed. The proximal surface of the radial head articular surface can be visualized (Fig. 8). To expose the remainder the radial head and neck an anterior release of the annular ligament is required. We only excise the radial head if there is preoperative localized tenderness, and pain with forearm rotation. If there is a lateral joint capsular contracture, then the lateral ligaments can be subperiosteally released from the lateral humeral ridge to improve visualization. However, this is not usually required.

#### Total elbow arthroplasty

**Figure 9:**
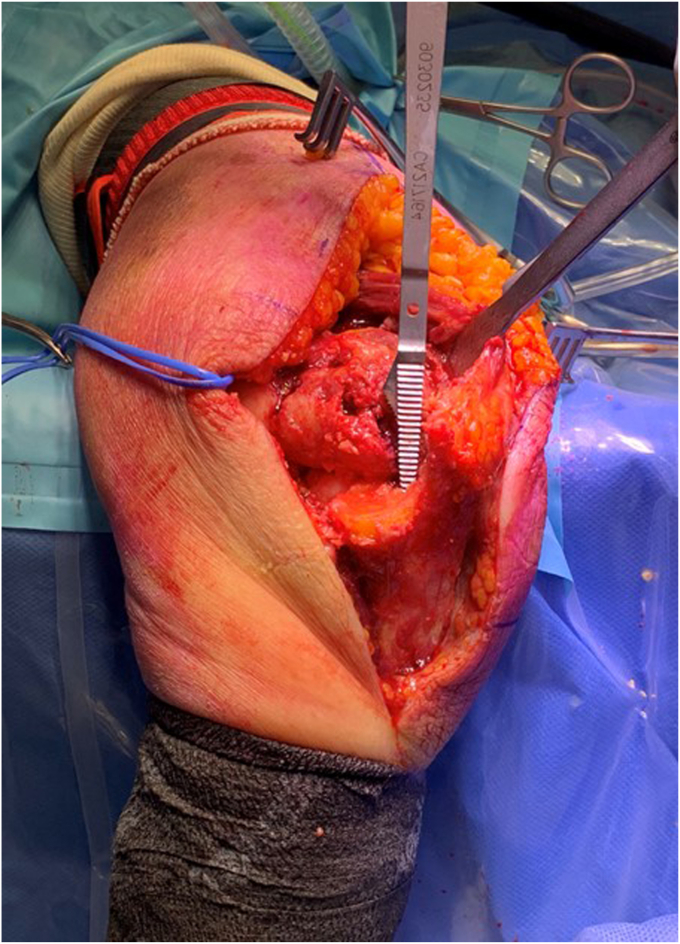
Clinical case with broaching of the ulnar shaft. Small Hohmann retractor is positioned to maintain exposure of the proximal ulna. Blue marker on the ulnar nerve. Copyright Dr. Gregory Bain 2025.

**Figure 10:**
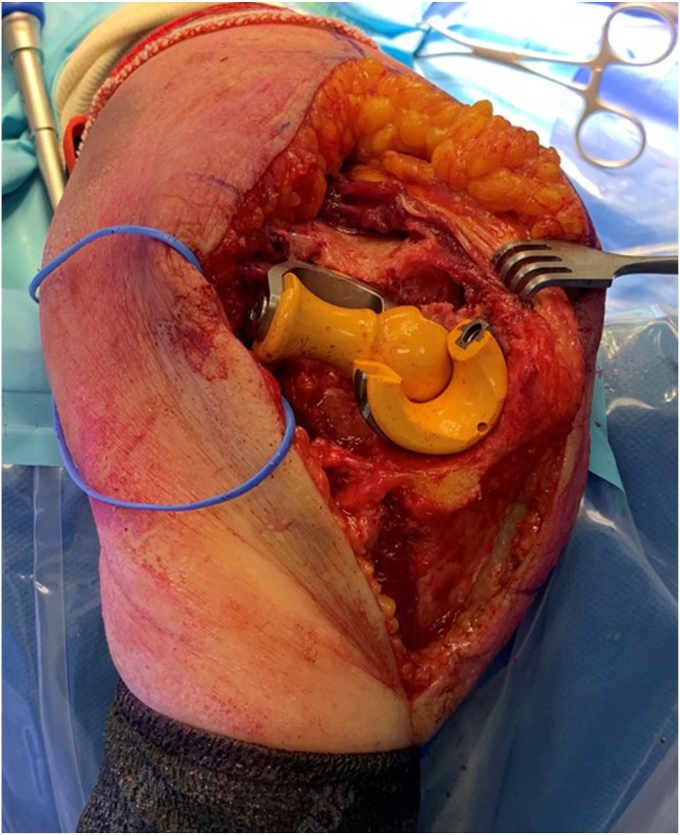
Following insertion of the humeral and ulnar trial implants. Note that the humerus and the ulna are orthogonal, hinging on the lateral ligament complex. Blue marker tape on the ulnar nerve. Copyright Dr. Gregory Bain 2025.

**Figure 11:**
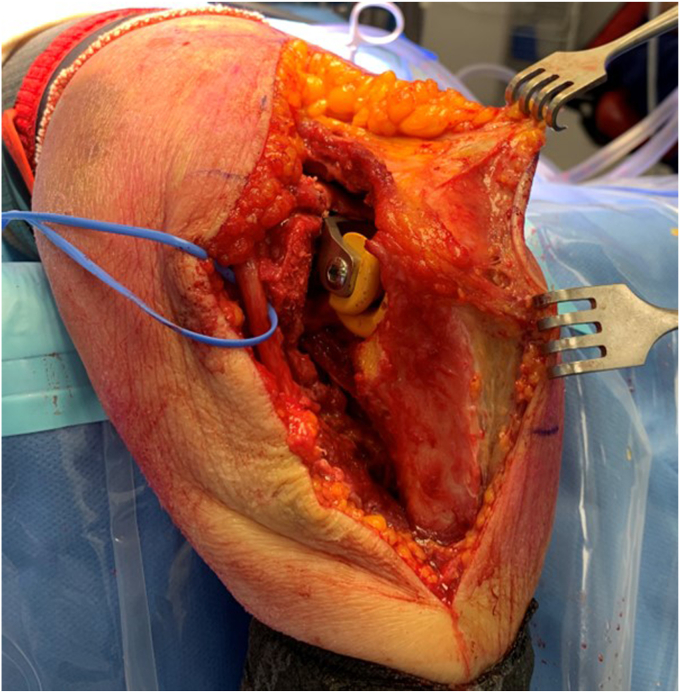
Reduction of the trial implants. Rack retractors on the skin, demonstrating that the olecranon and the olecranon bursa have not been exposed. Blue marker on the ulnar nerve. Copyright Dr. Gregory Bain 2025.

**Figure 12:**
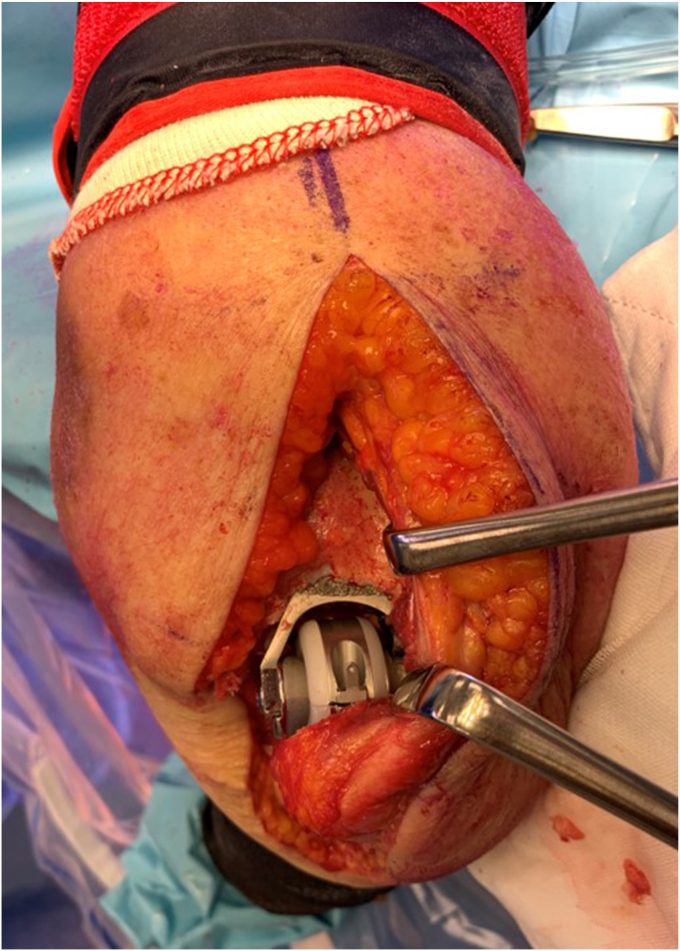
Definitive prosthesis insitu. The Langenbeck retractors exposing the distal humerus component and the articulation. The locking cap has been inserted via this exposure, with the appropriate screw. Copyright Dr. Gregory Bain 2025.

To perform a TEA, it is critical to have excellent exposure of the trochlear groove and coronoid process (Figure 9, Figure 10, Figure 11, Figure 12). A strategically placed Hohman retractor over the lateral olecranon, will assist in providing adequate exposure to enable visualization of the coronoid process, and thereby allows ulnar shaft instrumentation (Fig. 9). As the forearm is in an unusual orientation, it is important to assess the anatomical landmarks, before reaming the ulnar canal (Fig. 9). For orientation we palpate the flat posterior border of the proximal ulna, and observe the anterior coronoid process, medial sublime tubercle and lateral radial head.

Once the trial components are inserted, the elbow is taken through a range of motion. Initially this included a fluoroscopic assessment, to ensure an impingement free range of motion and reproduction of the center of rotation. Clear visualization of the coronoid process, ulnar component and anterior flange of the humeral component, is important to assess if there is anterior impingement, which can lead to ‘component pistoning’ and early failure^15^ (Figure 10, Figure 11, Figure 12).

#### Closure

**Figure 13:**
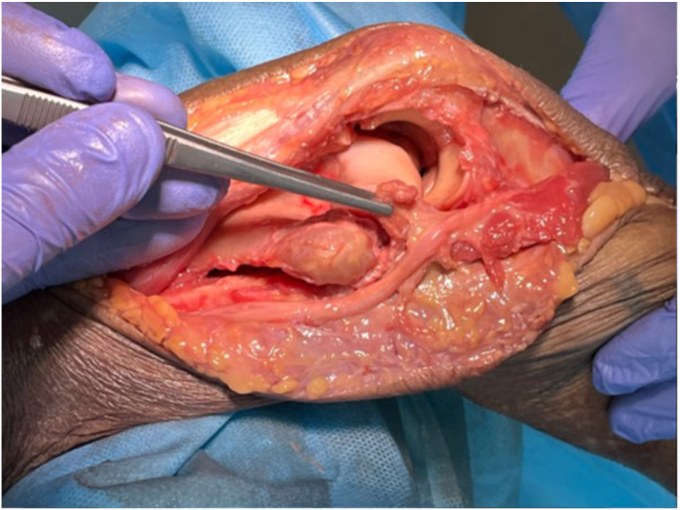
Joint reduction and MCL repair. The humerus was dislocated through the medial soft tissue window. Posterior to the humerus are the triceps and ulna. The anterior soft tissues include the brachialis, biceps, ulnar nerve, and common flexor origin muscles. The triceps attachment, olecranon, and its bursa tissue remain undisturbed. The MCL (forceps) is prepared for surgical stabilization. The common flexor origin would then be repaired to the medial epicondyle. Care is required to ensure the ulnar nerve is in a safe bed. Copyright Dr. Gregory Bain 2025. *MCL*, medial collateral ligament.

**Figure 14:**
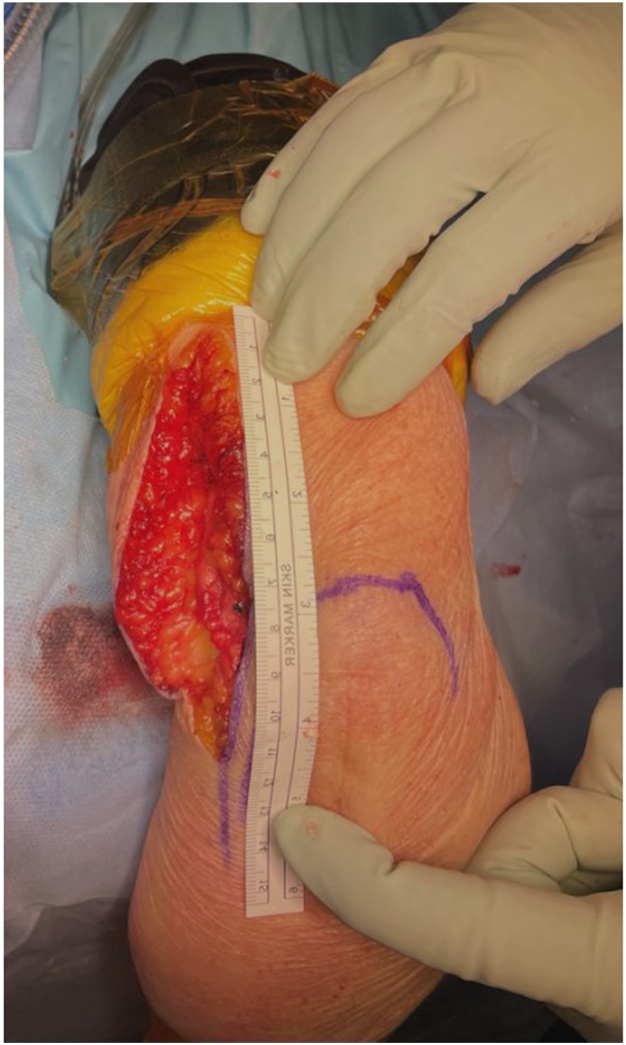
Prosthesis has been inserted, and the medial soft tissues repaired. Note that the incision is medial to the olecranon skin marking. Typically, the incision extends 10 cm proximal from the medial corner of the olecranon, and 5 cm distal. Copyright Dr. Gregory Bain 2025.

On the lateral side no internal or external repair or closure is required (Figs. 13 and 14). The exception is if a distal hemiarthroplasty is performed. In this case the capitellum has been excised, and sutures are placed into the lateral ligament complex. These sutures are shuttled through the cannulated screw within the anatomical spoon of the Latitude prosthesis.

The ulnar nerve is assessed to determine if it is in a safe bed, or if an anterior transposition is required. The sutures exiting the medial side of the screw, are placed into an eyed needle, and are secured to tissues on the medial side.^21^ The medial ligament complex (or medial epicondyle osteotomy) is securely repaired (Fig. 13). The common flexor origin is repaired to the medial epicondyle and FCU fascia to the proximal ulna. The wound is then closed in layers (Fig. 14).

#### Rehabilitation

As the incision is close to the mid-axial line, the patient is less likely to lean on the wound, and there is minimal tension on the incision throughout elbow motion. As the lateral ligament complex remain intact, and the medial ligament has been repaired, the joint is stable. As the insertion of the triceps, biceps and brachialis have not been violated, early active mobilization can safely be performed.

## Discussion

This technical report describes the development of a medial, single-window surgical approach to the elbow and its application in TEA. Cadaveric assessment demonstrates that this approach is feasible and provides adequate visualization of the articular surfaces necessary for insertion of an elbow arthroplasty. In our early clinical experience, we have performed this surgical approach safely and obtained exposure to insert the arthroplasty without compromising the alignment or stability of the prosthesis. A key advantage of this technique is the reduction in soft tissue dissection and dead space, by using a single window, avoiding violation of the olecranon bursa and lateral ligamentous structures. Additionally, the medial skin incision allows early postoperative motion with minimal tension across the wound, potentially reducing wound healing complications and postoperative pain.

The medial approach for arthroplasty has received limited attention within the literature. Prokopis et al^23^ describe a posterior midline incision with full thickness flaps laterally and medially. A lateral approach was required to deploy the linking pin. Therefore, the olecranon bursal tissue will be compromised and the benefits of a medially based incision on rehabilitation negated. Oizumi et al^17^ utilize a medially incision and took care not to disturb the tissue over the olecranon; however, they describe a wide ulnar nerve release and anterior transposition. The Selective Triceps-On Medial Paraolecranon approach has been recently reported, lending further credence to the new interest in medially based approaches.^22^ Although the humeral and ulnar instrumentation occurs through the medial window, a posterior midline incision is utilized, and an accessory lateral Kocher approach is required. The authors of the medial Selective Triceps-On Medial Paraolecranon approach do attest to the excellent visualization of the sigmoid notch, which they report to be a great improvement to the lateral paraolecranon approach.

Our technique, developed independently of these approaches, represents a further evolution—a purely medial single-window approach, that minimizes ulnar nerve dissection, often avoids the need for ulnar nerve transposition, maintains lateral ligament integrity, while achieving implant alignment using standard instrumentation. Finally, it facilitates early active rehabilitation and hopefully will reduce the infection rate as the olecranon bursa has not been violated.

### Preservation of the olecranon bursa

The olecranon bursa, a synovial membrane situated between the olecranon process and the overlying subcutaneous tissues and skin, is particularly vulnerable to inflammation and fluid accumulation following trauma or surgical intervention.^14^^,^^16^ The olecranon skin and subcutaneous tissues are very thin and depend upon a random pattern subcutaneous vascular plexus without any large perforators.^7^^,^^19^^,^^20^ Any incision through the skin and bursa carries a high risk of delayed healing, skin edge necrosis, sinus formation, and infection.^14^ Patients with gout, rheumatoid arthritis, diabetes, psoriasis, prior elbow surgery, or complex elbow injuries face an even greater risk.^4^ Mechanical factors such as swelling, direct pressure when leaning on the elbow, and the tension placed on a midline incision during flexion further jeopardize wound integrity. Even a small area of necrosis, with delayed wound healing, or wound dehiscence allows bursal fluid and/or hematoma to discharge, creating a sinus. If this localized defect does not heal, then the sinus will require urgent surgical closure or flap coverage to prevent a deep joint infection.^7^

By avoiding an incision and dissection of the olecranon skin and bursa, these wound healing complications and the risk of infection are significantly reduced. A medial incision lies over skin that receives a more robust blood supply from the subcutaneous plexus and fascial perforators.^7^ A medial incision is not placed under tension during elbow flexion. In addition, patients naturally avoid leaning on a medial wound, further protecting it from direct pressure.

### Triceps integrity and postoperative function

Triceps insufficiency imparts a significant functional limitation following elbow arthroplasty and may require complex revision surgery. A systematic review reported that triceps failure occurred in 3% of elbow arthroplasties.^12^ How the triceps is managed affects the incidence: triceps taken off the ulna (11% failure), triceps elevation (3%), triceps turndown (0.6%).^12^ Booker et al^5^ report no triceps failures from 532 ‘triceps-on’ approaches. This medial single-window approach adheres to this ‘triceps-on’ principle, allowing for early functional motion and rehabilitation.

### Ulnar nerve management

Ulnar nerve injury is a well-recognized complication of elbow arthroplasty, with a reported rate of 6%.^25^^,^^27^^,^^28^ Transient ulnar nerve symptoms of less than 6 weeks have been reported in up to 21% of patients.^8^ In this medial approach, the dissection is focused on the medial border of the triceps, and subcutaneous border of the ulna. The nerve is not directly dissected but is released indirectly with an inside/out release technique. The nerve stays within its soft tissue envelope, which minimizes the dissection and traction on the nerve, even during the ‘shotgun’ maneuver.

### Medial antebrachial cutaneous nerve considerations

Painful neuroma of the medial antebrachial cutaneous nerve is a recognized complication for medial elbow surgery. The posterior branch of the medial antebrachial cutaneous nerve, passes anterior to the medial epicondyle, and then passes more posterior, as it extends more distally.^13^ As our incision is posterior to the medial epicondyle and then extends 5 cm over the ulnar border of the olecranon, the posterior branch of the medial antebrachial cutaneous nerve is unlikely to be injured. Performing the incision with the elbow in 90° flexion, increases the safety margin.

### Surgical exposure and implant alignment

Correct implant positioning is critical for optimal functional outcomes and to reduce the risk of implant loosening and subsequent revision surgery.^6^^,^^11^ It has been postulated that ‘triceps-on’ approaches, where adequate exposure may be technically more challenging, are associated with a higher risk of implant malalignment. However, in the only published comparative cadaveric study assessing implant alignment between ‘triceps-on’ and ‘triceps-off’ techniques, no statistical differences were observed.^10^

Although not formally assessed within this study, the surgical approach was deemed adequate for correct implant alignment. Within the clinical cases, visual and on table radiographic assessment did not identify any concern regarding implant alignment. Further studies are warranted to quantitatively assess this factor in a larger patient cohort.

### Application for hemiarthroplasty and unlinked elbow arthroplasty

When performing a distal humeral hemiarthroplasty, or unlinked TEA, the integrity and/or repair of the collateral ligaments is paramount to the loading, performance and survival of the implant.^9^ This medial approach maintains the lateral ligament complex, and the medial ligaments are repaired, thereby providing stability. The dynamic stabilizers are maintained—triceps, brachialis, biceps, lateral muscle, and the medial muscles are repaired.

### Challenges and limitations

Adopting a new surgical approach presents unique challenges, even for experienced surgeons, particularly when incorporating novel technical concepts. It is important to appreciate that to achieve an adequate exposure necessitates a total release of the capsular attachments from the posterior, medial and anterior aspects of the distal humerus. This significant release is essential for dislocating the distal humerus from the trochlear fossa. Then the altered orientation of the humerus and ulna demands careful attention by the surgeon.

Unlike other approaches, the lateral ligament complex is typically preserved; however, in hemiarthroplasty, it must be repaired and stabilized, and in cases where the lateral joint space is tight, release of the lateral ligaments from the distal humerus may be required to create a balanced prosthetic articulation. Additionally, triceps-on techniques pose challenges in inserting the prosthesis and securing the articulation, necessitating extra care when reducing the joint and inserting the locking cap. The authors have successfully performed this procedure using the Latitude hemi and total arthroplasties (Stryker, Kalamazoo, MI, USA). We have not used other prostheses since developing this technique and therefore cannot comment specifically on the utility of this approach using other prosthetic locking mechanisms. Given these procedural modifications and potential difficulties, we recommend that surgeons first practice the exposure on a cadaveric specimen or surgical training model and use intraoperative fluoroscopy in early cases to ensure precise reaming and implant positioning.

This surgical approach has been developed through cadaveric assessment and has been used by the senior author in 10 clinical cases. The authors' believe this approach is safe and replicable, and anecdotal reports from therapists are positive regarding the reduction in early postoperative pain and a consequential ease in mobilization. The approach has been developed for elbow arthroplasty. We have applied this technique to manage complex distal humerus fractures, performing either hemiarthroplasty or TEA as needed. It has also proven effective in treating medial condyle distal humerus fractures, as well as in revision instability and arthroplasty cases.

## Conclusion

This study describes the cadaveric development of a new medial single window, triceps-on, olecranon bursa sparing elbow approach. It provides excellent exposure, allows the surgeon to safely manage the ulnar nerve and stabilize the joint. It avoids violating the olecranon bursa, which is likely to reduce the devastating wound healing complications and infections.

## Disclaimers

Funding: No funding was disclosed by the authors.

Conflicts of interest: The authors, their immediate families, and any research foundations with which they are affiliated have not received any financial payments or other benefits from any commercial entity related to the subject of this article.

## Supplementary data

Video 1 Video demonstrating the release of the soft tissues from the posterior, medial, and anterior aspects of the distal humerus. This allows the medial shotgun dislocation of the elbow. Copyright Dr. Gregory Bain 2025.

Video 2 Video demonstrating the medial window exposure and implantation of a total elbow prosthesis in a cadaveric specimen. Copyright Dr. Gregory Bain 2025.

## References

[1] Alonso-Llames M. (1972). Bilaterotricipital approach to the elbow: its application in the osteosynthesis of supracondylar fractures of the humerus in children. Acta Orthop Scand.

[2] Bain G.I., Mehta J.A. (2002). Operative treatment of elbow injuries.

[3] Bain G., Metha J., Lim Y. (2007). Advanced reconstruction: Elbow.

[4] Blackwell J.R., Hay B.A., Bolt A.M., Hay S.M. (2014). Olecranon bursitis: a systematic overview. Shoulder Elbow.

[5] Booker S.J., Smith C.D. (2017). Triceps on approach for total elbow arthroplasty: worth preserving? A review of approaches for total elbow arthroplasty. Shoulder Elbow.

[6] Brinkman J.M., De Vos M.J., Eygendaal D. (2007). Failure mechanisms in uncemented Kudo type 5 elbow prosthesis in patients with rheumatoid arthritis: 7 of 49 ulnar components revised because of loosening after 2–10 years. Acta Orthop.

[7] Bunkis J., Ryu R.K., Walton R.L., Epstein L.I., Vasconez L.O. (1985). Fasciocutaneous flap coverage for periolecranon defects. Ann Plast Surg.

[8] Dwyer T., Henry P.D., Cholvisudhi P., Chan V.W., Theodoropoulos J.S., Brull R. (2015). Neurological complications related to elective orthopedic surgery: part 1: common shoulder and elbow procedures. Reg Anesth Pain Med.

[9] Evans J.P., Evans J.T., Mohammad H.R., Sayers A., Blom A.W., Whitehouse M.R. (2022). How long does an elbow replacement last? A systematic review and meta-analysis of case-series and national registry reports with more than 10 years of follow-up. Acta Orthop.

[10] King A., Booker S., Thomas W.J., Smith C.D. (2018). Triceps on, alignment off? A comparison of total elbow arthroplasty component positioning with a triceps-on and a triceps-off approach. Ann R Coll Surg Engl.

[11] Lenoir H., Micallef J.P., Djerbi I., Waitzenegger T., Lazerges C., Chammas M. (2015). Total elbow arthroplasty: influence of implant positioning on functional outcomes. Orthop Traumatol Surg Res.

[12] Little C., Graham A., Carr A. (2005). Total elbow arthroplasty: a systematic review of the literature in the English language until the end of 2003. J Bone Joint Surg Br.

[13] Manoukov Y., Herisson O., Sali E., Sautet A., Masquelet A.C., Cambon-Binder A. (2020). Anatomy of the posterior branch of the medial antebrachial cutaneous nerve: a cadaveric study. Orthop Traumatol Surg Res.

[14] Mehta J.A., Bain G.I. (2004). Surgical approaches to the elbow. Hand Clin.

[15] Morrey M.E., Songy C., Triplet J.J., Cheema A.N., O’Driscoll S.W., Sanchez-Sotelo J. (2022). Unexpected high early failure rate of the Nexel total elbow arthroplasty. JSES Int.

[16] Nchinda N.N., Wolf J.M. (2021). Clinical management of olecranon bursitis: a review. J Hand Surg.

[17] Oizumi N., Suenaga N., Yoshioka C., Yamane S. (2015). Triceps-sparing ulnar approach for total elbow arthroplasty. Bone Joint J.

[18] Patterson S.D., Bain G.I., Mehta J.A. (2000). Surgical approaches to the elbow. Clin Orthop Relat Res.

[19] Pearl R.M., Johnson D. (1983). The vascular supply to the skin: an anatomical and physiological reappraisal—Part I. Ann Plast Surg.

[20] Pearl R.M., Johnson D. (1983). The vascular supply to the skin: an anatomical and physiological reappraisal—Part II. Ann Plast Surg.

[21] Phadnis J., Watts A.C., Bain G.I. (2016). Elbow hemiarthroplasty for the management of distal humeral fractures: current technique, indications and results. Shoulder Elbow.

[22] Prada C., Khan S., Goetz T., Alolabi B. (2025). Description of a new surgical approach for elbow arthroplasty: selective Triceps-On medial paraolecranon (STOMP) approach. JSES Int.

[23] Prokopis P.M., Weiland A.J. (2008). The triceps-preserving approach for semiconstrained total elbow arthroplasty. J Shoulder Elbow Surg.

[24] Rydholm U., Ljung P. (2003). Surface replacement of the rheumatoid elbow through a lateral approach. Tech Orthop.

[25] Stoddart M.T., Panagopoulos G.N., Craig R.S., Falworth M., Butt D., Rudge W. (2024). A systematic review of the treatment of distal humerus fractures in older adults: a comparison of surgical and non-surgical options. Shoulder Elbow.

[26] Studer A., Athwal G.S., MacDermid J.C., Faber K.J., King G.J. (2013). The lateral para-olecranon approach for total elbow arthroplasty. J Hand Surg.

[27] Voloshin I., Schippert D.W., Kakar S., Kaye E.K., Morrey B.F. (2011). Complications of total elbow replacement: a systematic review. J Shoulder Elbow Surg.

[28] Williams M.G., Donoghue S.J., Thomas E., Evans J.P., Thomas W., Smith C. (2024). The ‘vulnerable nerve’; surgically acquired neuropathy in distal humeral surgery patients and the role of a tourniquet. Shoulder Elbow.

